# Extrachromosomal circular DNA of multiple myeloma

**DOI:** 10.7150/jca.117290

**Published:** 2025-10-01

**Authors:** Fangfang Li, Xinyi Long, Sishi Tang, Jing Liu, Yunfeng Fu

**Affiliations:** 1 Department of Hematology, The Third Xiangya Hospital, Central South University, Changsha, 410013, China.; 2 Department of Blood Transfusion, The Third Xiangya Hospital, Central South University, Changsha, 410013, China.

**Keywords:** Multiple myeloma, Extrachromosomal circular DNA (EccDNA), Circle-seq, del (17p), Heterogeneity, CNP

## Abstract

**Background:** Extrachromosomal circular DNA (EccDNA) is widespread in various heterogeneous tumors and closely associated with tumor resistance and progression.

**Methods:** Circle-seq and mRNA-seq were done on samples from three multiple myeloma (MM) patients, one when they had a complete response and one when they relapsed.

**Results:** A large number of EccDNA molecules were detected with high heterogeneity among the six samples. Circle-seq combined with mRNA-seq analyses revealed that there is no linear relationship between mRNA expression and EccDNA quantity. Chromosome 19 presented the highest density of differentially expressed EccDNA genes, followed by chromosome 17. Only the T3 sample showed del(17p) by fluorescence *in situ* hybridization at the time of relapse. Approximately 92% of all upregulated EccDNA genes from chromosome 17 (137) were present in the T3 sample. By integrating Circle-seq and mRNA-seq data, we obtained several potentially functional candidate protein-coding genes and miRNAs from chromosome 17. We further assessed the prognostic value of the three protein-coding genes in the MMRF-COMPASS clinical trial and found that all three genes were poor prognostic indicators for MM. Furthermore, WB, CCK8 and Annexin V-FITC/PI assays revealed that the overexpression of 2',3'-cyclic nucleotide 3' phosphodiesterase (CNP) downregulated the apoptotic pathway and increased bortezomib tolerance in MM cells.

**Conclusion:** We performed a Circle-seq analysis of MM and investigated the heterogeneity of EccDNA. Analysis of data from clinical studies and basic experiments revealed that the gene carried on EccDNA most likely contributed to the increased tolerance of bortezomib in MM with del(17p).

## Introduction

Multiple myeloma (MM), a type of clonal plasma cell tumor, is the second most prevalent hematological cancer 1,2. The development of novel drugs such as proteasome inhibitors has prolonged the survival of patients with MM by up to 3-6 years. Although significant progress has been made in MM treatment strategies, the 10-year survival rate is only 17% 3. The clonal evolution of MM is eventually induced under various therapeutic pressures, ultimately resulting in drug resistance, disease progression, and relapse 4. Treatment is often challenging when the disease progresses to drug resistance. Hence, identification of the underlying mechanism of relapsed refractory MM is urgently needed to identify novel and effective treatment strategies.

Extrachromosomal circular DNA (EccDNA) is a group of circular DNA molecules that can be double- or single-stranded. These molecules vary in size and sequence, and they exist in the nucleus. They are derived from chromosomes, but they are independent of them 5,6. EccDNA was first confirmed in 1965, and research increased with the development of Circle-seq in 2015 7. EccDNA is widely distributed in eukaryotic organisms 5, including plants 7-9, ciliates 10, yeasts 11, xenopus 12, drosophila 13, mammals 14, and pigeons 15. Numerous physiological and pathological processes are impacted by EccDNA, as confirmed by several studies, including gene amplification, telomere length restoration, genome plasticity, and molecular sponges 5. Almost all the genes carried on EccDNA can be matched to human autosomes, indicating that EccDNA originates from autosomes rather than another independent set of genes. Although the mechanism of EccDNA production is still unclear, four widely accepted hypothesis models exist: the breakage-fusion-bridge (BFB) cycle, chromothripsis, episome model, and translocation-deletion-amplification model 5,16. In conclusion, EccDNA is the product of a highly unstable genome. EccDNAs can be categorized into five types according to their size and sequence: small polydispersed DNA (spcDNA), microDNA, t-circle/c-circle, ERC, and EcDNA. Tumor cells are the main source of microDNA and EcDNA. MicroDNA can transcribe noncoding RNAs and act as regulators in a series of biological processes, whereas EcDNA is large enough to carry complete genes, thus participating in oncogene amplification and genomic heterogeneity 5. Yi et al. established the CRISPR-based ecTag method to tag EcDNA with fluorescent markers in living cells and reported an uneven distribution of EccDNA during mitosis 17. EccDNA does not have centromeres; therefore, EccDNA is unevenly distributed in offspring cells during cell division, mediating intercellular heterogeneity. EccDNA has been studied in various highly heterogeneous tumors and is involved in tumor progression and drug resistance 18-23. EccDNA carrying oncogenes is associated with gastric cancer carcinogenesis and participates in gastric cancer progression 20. DNMT1^circle10302690-10302961^ is associated with metastasis and poor prognosis in advanced high-grade serous ovarian cancer 21. Amplification of the RAB3B-encoding gene on EccDNA leads to high expression of RAB3B, which induces cisplatin resistance in hypopharyngeal squamous cell carcinoma through autophagy 19. High expression of PLCG2 is caused by the amplification of the PLCG2-encoding gene on EccDNA, and metastasis of non-small cell lung cancer is mediated by this high expression through enhanced mitochondrial respiration 22. Amplification of the KIF3C-encoding gene on EccDNA downregulates MUC20, which weakens copper-mediated cell death, thereby inducing proteasome inhibitor resistance in multiple myeloma 24. Zou et al. synthesized DNA circles harboring the miR-17-92 cluster and verified that artificial DNA circles harboring the miR-17-92 cluster can significantly promote the proliferation and promotion of hepatocellular carcinoma 25. Paulsen et al synthesized microDNA molecules mimicking known microDNA sequences and discovered that they could create short regulatory RNA molecules that can repress gene expression without relying on the usual promoters 26. It is evident that the efficient transcription of coding genes carried on EccDNA is essential for EccDNA to function in tumors.

The differential expression of EccDNA in completely relieved and relapsed MM was investigated to identify the mechanisms and potential therapeutic targets of EccDNA in relapsed refractory MM. Therefore, we first explored the differential expression profiles of EccDNA between completely relieved and relapsed MM. We found that EccDNA heterogeneity was present not only between groups but also among the six samples. We also found that chromosome 19 had the highest density of differentially expressed EccDNA genes, followed by chromosome 17. Among the six samples, only T3 was del(17p), according to fluorescence in situ hybridization. Among all the upregulated differentially expressed EccDNA genes from chromosome 17 (137) in the six samples, approximately 92% were present in the T3 sample. We further integrated Circle-seq and mRNA-seq data and obtained several potentially functional candidate protein-coding genes and miRNAs from chromosome 17. Further analysis revealed that these candidate genes were located on three EccDNA molecules, with two being EcDNA and one being microDNA. However, EcDNA is too long to be synthesized. To further explore the function of EccDNA, we first assessed the prognostic value of the three-candidate protein-coding genes in the MMRF-COMPASS clinical trial and then selected CNP for WB, CCK8, and Annexin V-FITC/PI assays. We found that all three-candidate protein-coding genes were poor prognostic indicators for MM and that the overexpression of CNP increased bortezomib resistance in MM cells.

## Materials and Methods

### Patient selection and sample collection for sequencing

Due to the large volume of bone marrow samples required during remission and the fact that samples taken at complete remission and at relapse were obtained from the same patient, the samples were not purified for plasma cells. The effect evaluation of MM patients hospitalized at Xiangya Third Hospital of Central South University was performed according to the International Myeloma Working Group (IMWG) response criteria 27. When patients achieve complete remission and when they relapse, we collect bone marrow samples, further collect bone marrow mononuclear cells, and immediately transfer them to a -80°C freezer. The study was conducted with the patients' written informed consent and the approval of the Xiangya Third Hospital of Central South University's Ethics Committee.

### Cell lines

Four human MM cell lines—NCI-H929, RPMI 8226, ARP1, and U266—were procured from ATCC (Manassas, VA, USA). These cells were cultivated in RPMI 1640 medium (Basal Media, Shanghai, China), which was supplemented with 10% fetal bovine serum (FBS) (Sigma-Aldrich, St. Louis, MO, USA), 100 U/mL penicillin-streptomycin (100X) (New Cell & Molecular, Suzhou, China). The cells were cultivated in a humidified incubator with 5% CO2 at 37 °C. Both cell lines were authenticated via short tandem repeat (STR) profiling within the last three years, and cells free from mycoplasma were utilized in all experiments.

### EccDNA purification, circle-seq, data analysis

Previous reports have described how to purify, roll-circle amplify, and sequence EccDNA 28,29. Briefly, we used the QIAamp DNA Mini Kit (USA) to extract genomic DNA from bone marrow samples, followed by DNA quality control using a Nanodrop 1000 instrument and agarose gel electrophoresis. PacI restriction enzyme (New England Biolabs, USA) was used to linearize mitochondrial DNA (mtDNA). phenol/chloroform/isoamyl alcohol (PCI) solution (25:24:1) (Solarbio, Beijing, China) was used for DNA extraction. Phi29 DNA polymerase (New England Biolabs, USA) was used for rolling circle amplification (RCA). The Bioruptor® apparatus (Diagenode, Belgium) was utilized to shear amplified EccDNA to an average fragment size of 400 base pairs (bp). The NEBNext® Ultra™ II DNA Library Prep Kit (New England Biolabs, USA) was employed for library preparation for the EccDNA-seq. The Illumina cBot system and NovaSeq 6000 S4 kit (300 cycles) (USA) were used for library clustering. The Illumina NovaSeq 6000 platform was utilized in the generation of raw sequencing data. In order to trim the QC-filtered raw reads, the fastp software, version 0.23.2, was utilized. The Bioinformatics Workflow Advisor (BWA) software version 0.7.17 was utilized to perform alignment with the human genome (UCSC HG38). The detection of EccDNA was facilitated by the utilization of the Circle-Map V1.1.4 software, which was employed to analyze the sequencing data. A comparison of the differences in EccDNA genes between sample groups was conducted using total base pair evidence normalization (fragments per million: FPM). The cut-off criteria for identifying differentially expressed EccDNAs was established as follows: a |log2FoldChange| value of at least 1 and a p-value less than 0.05. In the event that a valid p-value was not obtained, the criteria were adjusted to |log2FoldChange| ≥ 3.

### mRNA enrichment, mRNA-seq, and data analysis

TRIzol reagent (Invitrogen, Carlsbad, California, USA) was utilized to extract total RNA from bone marrow samples. The quality of the extracted RNA was subsequently assessed using a Nanodrop 1000 instrument and agarose gel electrophoresis. The Next® Poly(A) mRNA Magnetic Isolation Module (New England Biolabs, USA) was utilized for the purpose of enriching mRNA. The preparation of mRNA sequencing libraries was undertaken with the utilization of the KAPA Stranded RNA-Seq Library Prep Kit (Roche, Switzerland). The Agilent 2100 Bioanalyzer (USA) was utilized in the evaluation of the quality of the constructed libraries. The NovaSeq 6000 S4 Kit (300 cycles) (Illumina, USA) was employed for in situ dilution and amplification of the prepared libraries. The Illumina NovaSeq 6000 (USA) was used for sequencing. The fastp software (V0.23.2) was used for quality control of the raw sequence data. BWA software (V0.7.17) was used for alignment to the human genome (UCSC HG38). The quantification of sample expression levels, as well as the differential expression analysis of genes and transcripts between groups, were performed using the Ballgown R package. The screening thresholds for differentially expressed genes and transcripts were established as follows: a fold change of at least 1.5, a p-value of less than 0.05%, and an FPKM mean of at least zero for each group.

### Transfection

Sangon Biotechnology (Shanghai, China) supplied the CNP-overexpressing pcDNA3.1 plasmids and negative controls, and we used Lipo8000™ (Beyotime, Shanghai, China) for cell transfection in 12-well plates.

### RNA isolation, real-time quantitative reverse transcription‒PCR (RT‒qPCR)

RNAiso Plus (TaKaRa, Tokyo, Japan) was used to extract total RNA from MM cells. CNP and β-actin were quantified via HiScript II Q RT Super Mix for qPCR and ChamQ Universal SYBR qPCR Master Mix (Vazyme, Nanjing, China). The experiment was conducted on three separate occasions. The primers utilized were as follows: β-actin, forward: 5ʹ-GAGACCTTCAACACCCCAGC-3ʹ; reverse: 5ʹ-ATGTCACGCACGATTTCCC-3ʹ; and CNP, forward: 5ʹ-GAGTACGCTCAACAAGATGTGT-3ʹ; reverse: 5ʹ-AGCTTGTCCACATCACTCGG-3ʹ.

### Cell viability assay

The half-maximal inhibitory concentration (IC_50_) was determined via a Cell Counting Kit-8 (Abiowell, Changsha, China). 200 μL cell suspension (1×10^4^ cells/well) were seeded in a 96-well plate and treated with various doses of bortezomib (4-32 nmol/L) (MedChemExpress, USA). After 24 hours, each well was added with 20 µL of CCK-8 solution. After incubating for two hours at 37°C, the absorbance was measured at 450 nm. The experiment was replicated thrice.

### Annexin V-FITC/PI assay

Annexin V-FITC/PI apoptosis detection kit (Vazyme, Nanjing, China) was used to analyze apoptosis (Vazyme, Nanjing, China). The cells were exposed to bortezomib (Med Chem Express, USA) at a concentration of 10 nmol/L for a duration of 24 hours. The evaluation of apoptotic cells was subsequently conducted and the subsequent analysis of the data was facilitated. We did the experiment thrice.

### Fluorescence *in situ* hybridization (FISH)

The analysis of CD138-purified plasma cells was conducted using a fluorescence in situ hybridization (FISH) approach, employing the p53/D13S319 probe (provided by HealthCare Biotechnology, Wuhan, China). In each analysis, a minimum of 200 interphase nuclei were considered, and an abnormal threshold of 10% was established.

### Western blotting (WB)

The cell lysis buffer (Beyotime, Shanghai, China) was used to lyse the cells after collection for WB analysis, with the buffer containing 10 nmol/L protease and phosphatase inhibitors. The bands were visualized via enhanced chemiluminescence (ECL) reagent (Absin, Shanghai, China). An anti-caspase-3 antibody was purchased from Cell Signaling Technology (Danvers, MA, USA). The anti-β-actin and HRP-conjugated goat anti-rabbit IgG antibodies were obtained from ABclonal in Wuhan, China.

### Statistical analysis

The data are expressed as the means ± SDs of at least three independent experiments. The statistical significance of the differences was evaluated via a student's t test using GraphPad Prism software (Prism 10). Statistical significance was set at P < 0.05.

## Results

### Characterization of MM patients and EccDNA expression in MM

**Figure 1:**
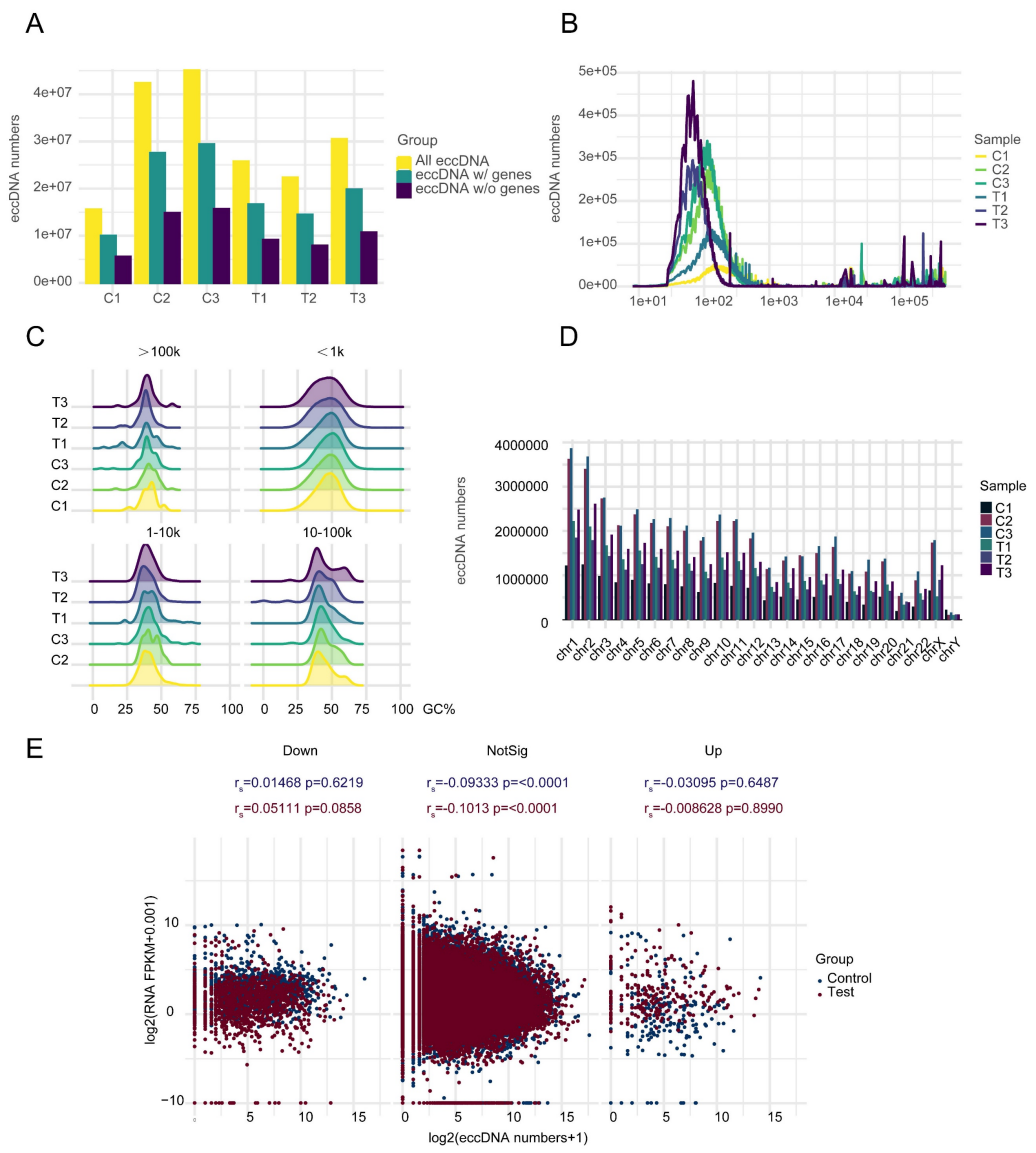
EccDNA profile in MM. A Numbers of total EccDNA molecules, EccDNA molecules with genes, and EccDNA molecules without genes detected in six samples. B Length distribution of EccDNA in six samples. C GC content distribution of EccDNA of different sizes in six samples. D EccDNA distribution on chromosomes in six samples. E Correlation analysis of mRNA expression and the number of EccDNA carrying the same gene in the subgroups in which mRNA was upregulated, not significantly different, or downregulated.

Basic information about three MM patients, including age, sex, monoclonal gammopathy type, treatment, and bone marrow at the time of recurrence has been reported in our previous article 30. After RNA and DNA samples pass quality control, the mRNA-seq and circle-seq processes are conducted. A large number of EccDNA molecules (341217-1148828) were detected in six samples, with more EccDNA-carrying genes than those not carrying genes (Figure 1A). Most of the EccDNAs were a few hundred bp in length, with lengths ranging from tens to millions of bp, as shown in Figure 1B. The GC content of the EccDNA molecules was approximately 50%. The larger the molecule is, the higher the GC content (Figure 1C). The EccDNA sequences were all aligned to 46 chromosomes, and the results revealed that the longer the chromosome was, the more EccDNAs there were (Figure 1D). Circle-seq combined with mRNA-seq analysis revealed that there is no linear relationship between mRNA expression and EccDNA quantity in either the mRNA upregulation group or the mRNA downregulation group, and in either the control group or the test group (Figure 1E).

### Differentially expressed EccDNA genes between the two groups

**Figure 2:**
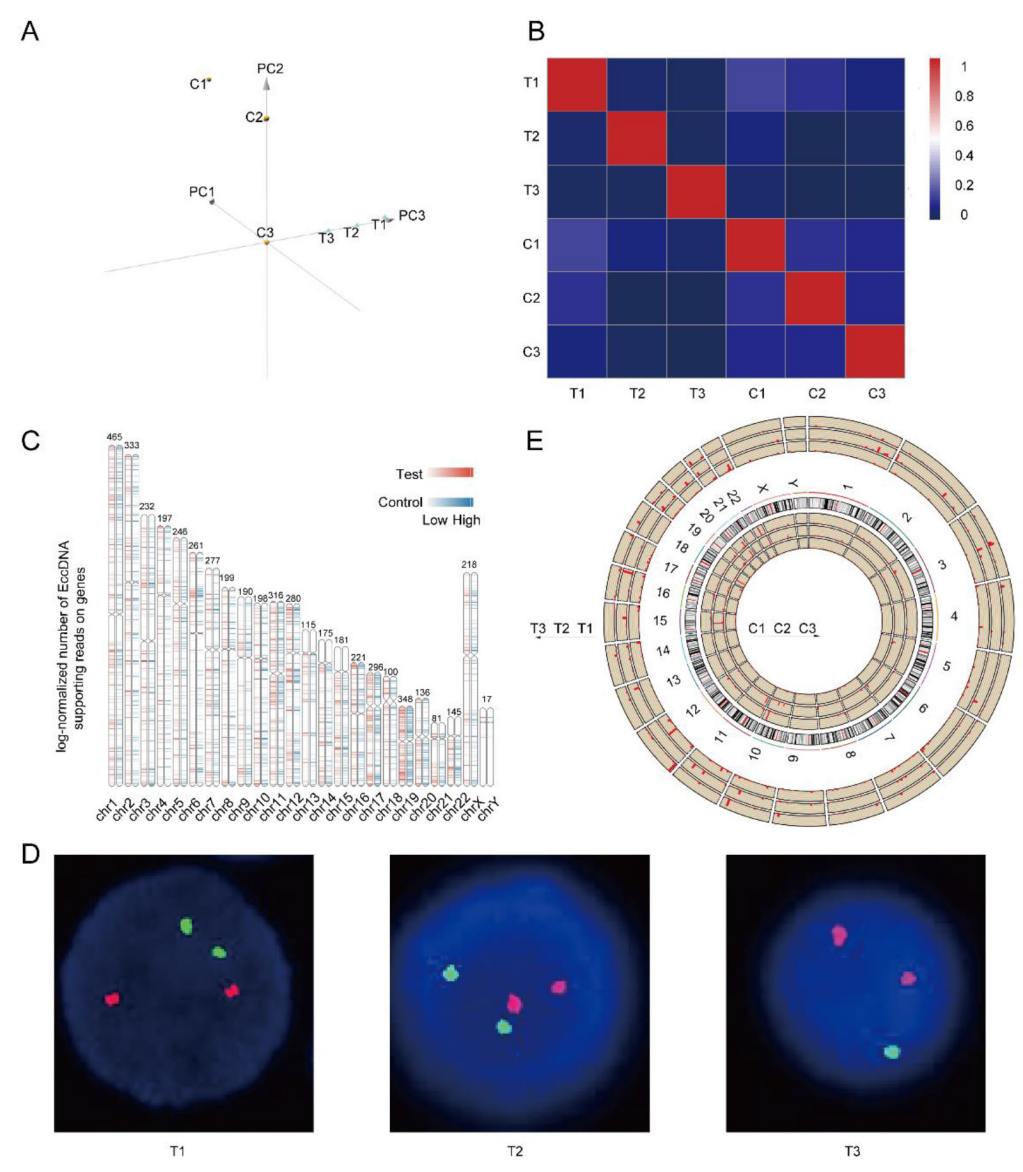
Heterogeneity in the EccDNAs of six samples and differentially expressed EccDNA genes between the two groups. A Principal component analysis (PCA) of six samples. B Correlation heatmap of six samples. C Differentially expressed EccDNA genes distribution on chromosomes. D p53/D13S319 gene deletion probe FISH images of three samples in the relapse group (FISH was not performed in the complete response group), with the D13S319 probe red and the p53 probe green. E Distribution of upregulated differentially expressed EccDNA genes on all chromosomes of all six samples, with the length of the red line indicating the number of upregulated differentially expressed EccDNA genes.

Both principal component analysis (PCA) and correlation heatmaps revealed obvious heterogeneity, not only between the completely relieved group and the relapsed group but also within the six samples, as shown in Figure 2A-B. As shown in Figure 2C, the differentially expressed EccDNA genes originated from all the chromosomes, with the Y chromosome having the fewest. Chromosome 19 had the highest density of differentially expressed genes, followed by chromosome 17. The clinical data of three patients were analyzed, and we found that the T3 sample was del(17p) by fluorescence in situ hybridization (FISH) (Figure 2D). However, no 17p deletion was detected in any of the other five samples (FISH is performed on purified myeloma cells. Because there are no myeloma cells in the bone marrow of patients in complete remission, there is no significance to perform FISH. As myeloma cells were present in the bone marrow of patients in relapse, FISH testing was performed to detect 17p deletion conventionally). The chromosomal distribution of upregulated differentially expressed genes in all six samples is shown in Figure 2E (& Supplementary Table 1). Approximately 91% of the upregulated differentially expressed EccDNA genes (2359 in total) were present in the T3 sample. Only 5% of the differentially expressed EccDNA genes in T3 originated from chromosome 17. However, approximately 92% of all upregulated differentially expressed EccDNA genes (137 in total) in all six samples were from chromosome 17 in T3.

### Upregulated differentially expressed EccDNA genes from chromosome 17

**Figure 3:**
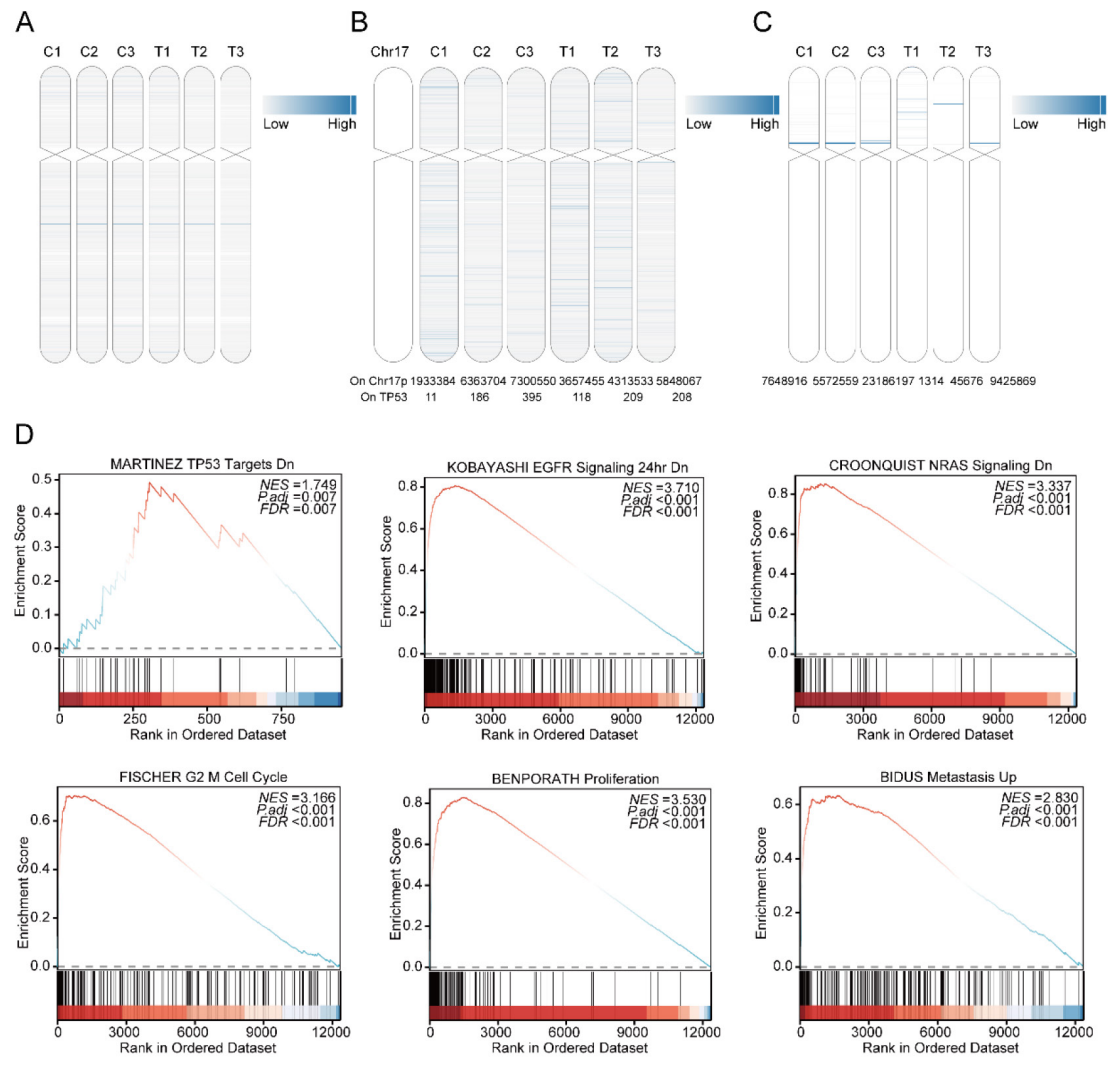
Upregulated differentially expressed EccDNA genes from chromosome 17. A mRNA expression levels of genes carried by EccDNA on chromosome 17 in six samples, with the depth of the blue line indicating the expression level of the mRNA. B Number of EccDNA junction sites located at 17p and TP53 in six samples. TP53 is located at the red line, with the depth of the blue line indicating the number of junction sites. The numbers below the picture are specific quantities. C The number of super enhancers carried by EccDNA located at 17p, with the depth of the blue line indicating the number of super enhancers. D GSEA enrichment analysis of upregulated differentially expressed EccDNA genes on chromosome 17 (test group vs control group).

As a recognized high-risk factor in multiple myeloma, del(17p) is present in approximately 10% of patients at first diagnosis and relapse 31. The gene expression of EccDNA on chromosome 17 in all six samples was determined via mRNA-seq and is shown in Figure 3A. All six samples had EccDNA genes from chromosome 17, and the relieved MM samples did not have fewer EccDNA genes than the relapsed MM samples did. The extremely poor prognosis of MM patients with del(17p) may be due to the deletion of the TP53 gene 32. The number of EccDNA molecules whose junction site was located on chromosome 17, as well as whose junction site was located on the TP53 gene, in all six samples is shown in Figure 3B, clearly demonstrating that no significant predominance was observed in the T3 sample. As shown in Figure 3C, many super enhancer-harboring EccDNAs from chromosome 17 were detected in all six samples. As shown in Figure 3D, Upregulated differentially expressed EccDNA genes on chromosome 17 are enriched in TP53 target genes, and are enriched in the EGFR and NRAS pathways, which are closely related to tumorigenesis and development, as well as in cell cycle checkpoints, proliferation, and metastasis pathways. This is consistent with the poor prognosis associated with multiple myeloma with del(17p).

### EccDNA carries genes with potential functions

**Figure 4:**
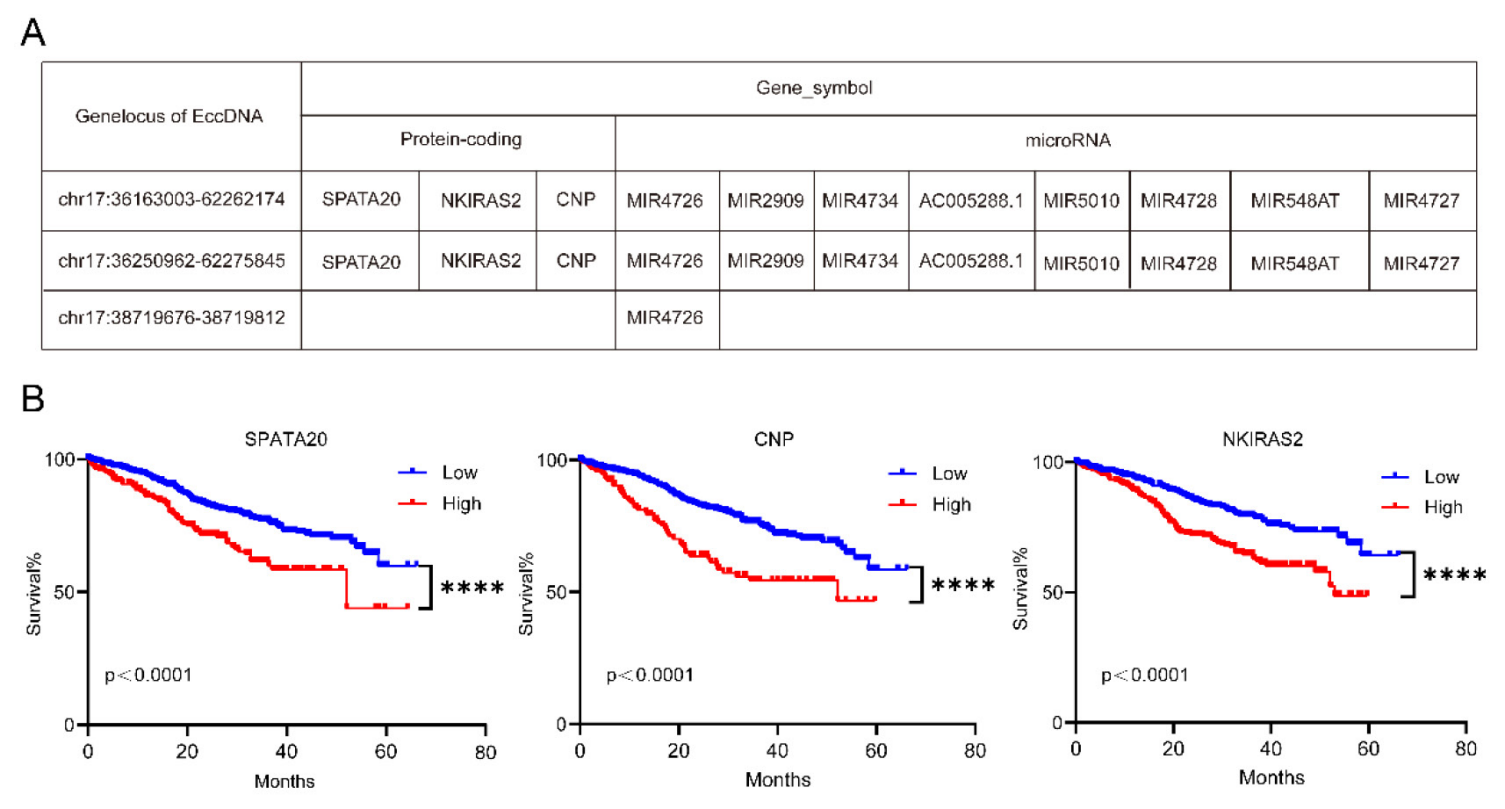
EccDNAs carry genes with potential functions. A Three EccDNA molecules and the candidate genes they carry. B Overall survival (OS) associated with the SPATA20, CNP and NKIRAS2 levels estimated in patients included in the MMRF-COMMPASS dataset. P values were calculated with a log-rank test.

Some candidate genes were obtained after taking the intersection of upregulated differentially expressed EccDNA-protein coding genes and upregulated differentially expressed mRNAs from chromosome 17 and after taking the intersection of upregulated differentially expressed EccDNA-small RNA genes and downregulated differentially expressed mRNAs from chromosome 17 (Supplementary Table 2-3). These candidate genes were localized to three EccDNA molecules, namely, EccDNA^chr17:36163003--62262174^, EccDNA^chr17:36250962--62275845^, and EccDNA^chr17:38719676--38719812^, with the first two being EcDNA and the latter being microDNA. The three EccDNAs and the genes they carry are shown in Figure 4A. We further focused on the protein-coding genes encoded by the EcDNA. We further tested the associations of these three protein-coding genes with clinical outcomes in the MMRF-COMMPASS dataset from the TCGA. We found that all three protein-coding genes were poor prognostic indicators of MM (Figure 4B).

### Overexpression of CNP increases bortezomib tolerance in MM cells

**Figure 5:**
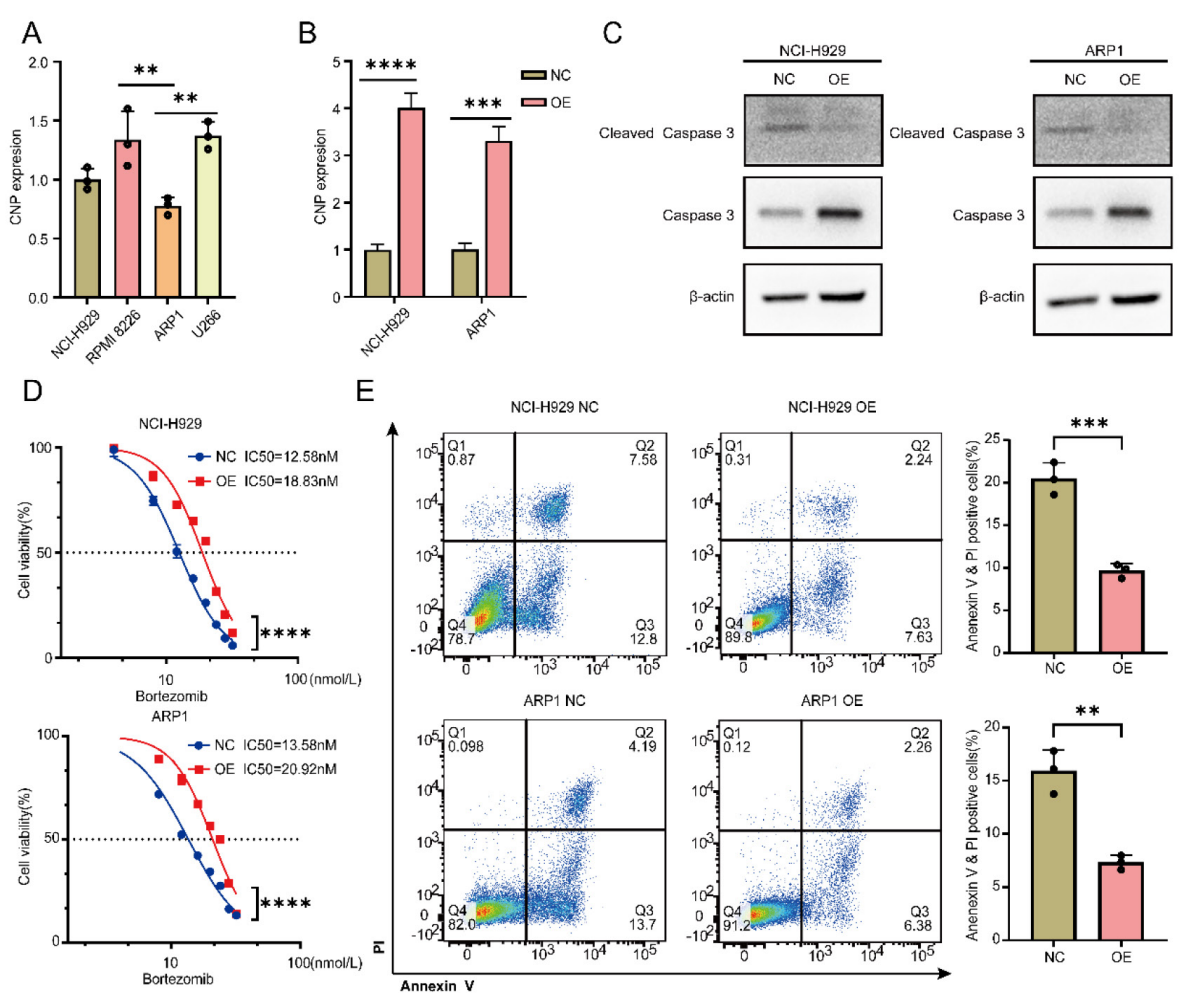
The overexpression of CNP increases the tolerance of MM cells to bortezomib. A qPCR analysis of CNP expression in 4 MM cell lines. B The expression of CNP in NCI-H929 cells and ARP1 cells was analyzed using qPCR after the cells were infected with OE-CNP plasmids or negative controls for 48 hours. C WB analysis of caspase 3 and cleaved caspase 3 expression was performed in NCI-H929 cells and ARP1 cells that had been transfected with OE-CNP plasmids or negative controls for 24 hours. The cells were then treated with bortezomib for an additional 24 hours. D The cell viability of the NCI-H929 and ARP1 cell lines was examined after they were transfected with OE-CNP plasmids or negative controls and then treated with different concentrations of bortezomib for 24 hours. E Apoptosis in NCI-H929 and ARP1 cells, as detected by the Annexin V-FITC/PI assay, after 24 hours of bortezomib treatment at 10 nmol/L, with OE-CNP plasmids or negative controls. The statistical significance of the test was determined using a student's t-test. *p<0.05, **p<0.01, ***p<0.001. **** p < 0.0001.

We wanted to synthesize EccDNA molecules for further experiments but failed because EcDNA is too long to loop. Moreover, EcDNA carry several genes that are not suitable for subsequent studies. To further verify that the genes carried on EccDNA were functional, we chose CNP for further studies. Four MM cell lines were found to express CNP, with the lowest levels of CNP being detected in NCI-H929 and ARP1 cells (Figure 5A). After successful transfection of the OE-CNP plasmids (Figure 5B), bortezomib was administered to NCI-H929 and ARP1 cells for a period of 24 hours. WB experiments were performed to detect the apoptotic pathway. As shown in Figure 5C, compared with control cells, both NCI-H929 and ARP1 cells overexpressing CNP presented a decrease in activated caspase 3. Furthermore, after 24 hours of bortezomib treatment, the IC50 increased in both NCI-H929 cells and ARP1 cells when CNP was overexpressed, in comparison to the negative control group (Figure 5D). Additionally, CNP overexpression decreased the proportion of apoptotic NCI-H929 and ARP1 cells (Figure 5E).

## Discussion

Many EccDNA molecules have been detected in MM, and our results are in agreement with earlier findings 18-23. The length distribution, chromosomal distribution, and high GC content of EccDNA molecules in MM are similar to those in other tumor types 18-23. There are more EccDNA molecules that carry genes (including longer protein coding genes and shorter non-coding genes such as lincRNA, microRNA) than not, suggesting that EccDNA molecules are produced to regulate gene expression. However, we further analyzed the mRNA expression and number of EccDNAs carrying concordant genes and found no correlation between mRNA expression and EccDNA, suggesting that only a few key EccDNA molecules are generated during the disease process and are involved in disease progression and drug resistance.

MM is a highly heterogeneous malignant plasma tumor with a survival period ranging from a few months to 10 years 33. Numerous studies have reported extensive inter- and intra-patient heterogeneity in patients with multiple myeloma at the genomic level 34-38, including single-cell RNA sequencing, whole-genome sequencing, and bisulfite sequencing. In this study, circle-seq analysis was performed on bone marrow samples from three MM patients when they achieve complete remission or when they relapse. The results also revealed high heterogeneity in all six samples. Importantly, the heterogeneity among the three samples in the relapsed group could be attributed to their different genetic abnormalities. However, there was still high heterogeneity among samples in the complete response group without any genetic abnormalities due to the tumor burden. The high heterogeneity of multiple myeloma is due to extremely frequent clonal evolution and highly unstable genomes 39,40. Quantitative multigene fluorescence in situ hybridization (QM-FISH) was performed in paired newly diagnosed and relapsed MM patients, and the clonal evolution of multiple myeloma was divided into four models 41, demonstrating that the clonal evolution of multiple myeloma is variable. We speculate that the evolution of MM clones was accompanied by the production of a large amount of EccDNA and that the noncoding RNA and protein-coding genes carried on these EccDNAs are functional, thereby mediating the high heterogeneity of MM. Genes on autosomal chromosomes can be transcribed only after the double helix structure of chromosomes is opened and the chromatin is depolymerized. However, EccDNA can be transcribed directly without restricting the opening of the double helix or depolymerization of the chromatin. Compared with autosomal genes, EccDNA can function more promptly and efficiently in response to environmental changes. Moreover, several studies have shown that large amounts of EccDNA are detected in normal human tissues and body fluids 42-44. In view of the above findings, we hypothesized that the EccDNA genome, like the autosomal genome, has individualized characteristics. “Good EccDNA” are essential for the survival of cells, and once “bad EccDNA” are produced, they may lead to various disorders.

All 46 chromosomes produced differentially expressed EccDNA genes; the shorter the chromosome was, the fewer differentially expressed EccDNA genes. The density of differentially expressed EccDNA genes was the highest on chromosome 19, followed by chromosome 17. Among the six samples, only T3 was del (17p). Considering the extremely poor prognosis of MM patients with del(17p), we further analyzed the differentially expressed EccDNA genes on chromosome 17. We noticed that only 5% of the differentially expressed EccDNA genes in the T3 sample originated from chromosome 17 and that the T3 sample accounted for 92% of all upregulated differentially expressed EccDNA genes originating from chromosome 17 in all six samples, suggesting that the upregulated differentially expressed EccDNA genes originating from chromosome 17 in the T3 sample may be involved in disease progression and disease resistance. Del(17p) is a high-risk factor for MM 45. Newly diagnosed MM patients with del(17p) are associated with shorter overall survival (OS) 46, as are patients who acquire del(17p) later during the disease course 47. Neither chemotherapy nor autologous hematopoietic stem cell transplantation has been shown to completely overcome the adverse prognosis of MM with del(17p) 32,48, even in the era of new drug therapies. Del (17p) has been demonstrated to be a significant predictor of disease progression and drug resistance 31,49,50. For a long time, TP53 loss of heterozygosity was believed to be responsible for the poor prognosis of MM patients with del (17p). However, there are approximately 500 genes on chromosome 17p, among which both new oncogenes and tumor suppressor genes have been discovered 51-54. Here, we speculated that del(17p) may have formed a large amount of EccDNA, so we wondered whether other genes from chromosome 17 are involved in the very poor prognosis of del(17p). It is also possible that many EccDNA junction sites are located in the TP53 gene, which leads to its deletion. Therefore, we focused mainly on the upregulated differentially expressed EccDNA genes on chromosome 17p. We further analyzed the junction sites on chromosome 17 and TP53 of the upregulated differentially expressed EccDNA in all six samples. Among them, neither junction sites on chromosome 17 nor TP53 in the T3 sample had the highest frequency of upregulated differentially expressed EccDNA. EccDNA functions as a mobile enhancer 55. Hence, we analyzed EccDNA carrying super enhancers derived from 17p and found that the T3 sample had a large number of super enhancer-carrying EccDNA but not most of the other six samples.

EccDNA downregulates MUC20 via KIF3C amplification, thereby mediating resistance to bortezomib in MM 24. In this investigation, some candidate genes were screened after the intersection of upregulated differentially expressed EccDNA-protein coding genes and upregulated differentially expressed mRNAs from chromosome 17 was identified and after the intersection of upregulated differentially expressed EccDNA-small RNA genes and downregulated differentially expressed mRNAs from chromosome 17 was identified. These results indicate that these potential genes might be linked to bortezomib resistance in MM patients with del(17p). These candidate genes are located on three EccDNA molecules: two EcDNA and one microDNA. As mentioned above, EcDNA are sufficiently large to carry complete genes and thus participate in oncogene amplification and genomic heterogeneity 5. Therefore, we wanted to synthesize EcDNA to further confirm its function in the cell; however, we failed because it was too long to form a loop. EcDNA carry several genes and are not ideal targets for research. More evidence is needed to support the involvement of EccDNA in bortezomib resistance. We focused on three candidate protein-coding genes and found that all were poor prognostic indicators for MM by analyzing the MMRF-COMPASS dataset. We further selected CNP for cell function experiments. The results revealed that the overexpression of CNP inhibited the apoptosis pathway, leading to an increase in the IC_50_ and a decrease in the apoptosis of MM cells after treatment with bortezomib. All the above results suggest that some of the genes carried by EccDNA may be highly expressed and thus involved in bortezomib resistance. Outward PCR and Sanger sequencing have been reported to identify the circular characteristics of EccDNA molecules in studies on EccDNA^19^-^21^,^29^,^44^. In the future, we will identify specific EccDNA on chromosome 17, which will facilitate further research.

Our study revealed that a small number of key EccDNA molecules were generated during disease resistance, progression, and relapse, rather than a large number, for the following reasons. First, many EccDNA molecules and differentially expressed EccDNA genes remained in the complete remission group. Second, in both the complete remission group and the relapse group, whether in the mRNA upregulation group or the downregulation group, there is no correlation between mRNA expression and EccDNA quantity. Third, the samples without del (17p) still had many EccDNA with junction sites at 17p, TP53 and EccDNA carrying super enhancers originating from 17p.

## Conclusion

In conclusion, we first investigated and characterized the role of EccDNA in complete response MM and relapsed MM via circle-seq analysis. Our findings highlight that the heterogeneity of EccDNA is consistent with the heterogeneity of MM and may be a driver of the heterogeneity of MM. Moreover, we found that only some key EccDNA molecules may be involved in disease resistance, progression, and relapse by analyzing circle-seq data in combination with mRNA-seq data. Analyses of MM datasets in public databases, as well as functional experiments, have revealed that some key EccDNA molecules may be involved in bortezomib resistance. However, this investigation was conducted on three samples, and the sample volumes were limited. A larger sample size would pave the way for further confirmation and discovery of novel biomarkers. Moreover, we failed to synthesize EccDNA, and more direct evidence of EccDNA function is needed.

## Supplementary Material

Supplementary table 1: sig_diff_TvsC_ecc.

Supplementary table 2: Overlapping_Result 1.

Supplementary table 3: Overlapping_Result 2.

## Abbreviations

EccDNA: Extrachromosomal circular DNA
MM: Multiple myeloma
CNP: 2',3'-cyclic nucleotide 3' phosphodiesterase
BFB: Breakage-fusion-bridge
spcDNA: Small polydispersed DNA
IMWG: International Myeloma Working Group
FBS: Fetal bovine serum
STR: Short tandem repeat
RCA: Rolling Circle Amplification
mtDNA: Mitochondrial DNA
PCI: Phenol/chloroform/isoamyl alcohol
IC_50_: Half maximal inhibitory concentration
FPM: Fragments per million
FISH: Fluorescence *in situ* hybridization
WB: Western blotting
PCA: Principal component analysis
QM-FISH: Quantitative multigene fluorescence *in situ* hybridization
OS: Overall survival
BM: Bone marrow morphology
